# Characteristics of Volatile Organic Compounds Emitted from Airport Sources and Their Effects on Ozone Production

**DOI:** 10.3390/toxics12040243

**Published:** 2024-03-26

**Authors:** Mubai Chen, Shiping Li, Long Yun, Yongjiang Xu, Daiwei Chen, Chuxiong Lin, Zhicheng Qiu, Yinong You, Ming Liu, Zhenrong Luo, Liyun Zhang, Chunlei Cheng, Mei Li

**Affiliations:** 1 Institute of Mass Spectrometry and Atmospheric Environment, Guangdong Provincial Engineering Research Center for Online Source Apportionment System of Air Pollution, Jinan University, Guangzhou 510632, China; cmb123@stu2021.jnu.edu.cn (M.C.); xuyongjiang@stu2022.jnu.edu.cn (Y.X.); chendv@stu2022.jnu.edu.cn (D.C.); yyn123@stu2021.jnu.edu.cn (Y.Y.); 2 Shenzhen Ecological and Environmental Monitoring Center of Guangdong Province, Shenzhen 518049, China; 13602637069@163.com (S.L.); yunlszemc@126.com (L.Y.); lin.c.x@163.net (C.L.); qzcclass@163.com (Z.Q.); 3 Guangzhou Hexin Instrument Co., Ltd., Guangzhou 510530, China; lm3.1415@hotmail.com (M.L.); 17303027827@163.com (Z.L.); nbzly123456@163.com (L.Z.); 4 Guangdong-Hongkong-Macau Joint Laboratory of Collaborative Innovation for Environmental Quality, Guangzhou 510632, China

**Keywords:** volatile organic compounds, oxygenated volatile organic compounds, airport source, ozone formation potential

## Abstract

In recent years, commercial air transport has increased considerably. However, the compositions and source profiles of volatile organic compounds (VOCs) emitted from aircraft are still not clear. In this study, the characteristics of VOCs (including oxygenated VOCs (OVOCs)) emitted from airport sources were measured at Shenzhen Bao’an International Airport. The results showed that the compositions and proportions of VOC species showed significant differences as the aircraft operating state changed. OVOCs were the dominant species and accounted for 63.17%, 58.44%, and 51.60% of the total VOC mass concentration during the taxiing, approach, and take-off stages. Propionaldehyde and acetone were the main OVOCs, and dichloromethane and 1,2-dichloroethane were the main halohydrocarbons. Propane had the highest proportion among all alkanes, while toluene and benzene were the predominant aromatic hydrocarbons. Compared with the source profiles of VOCs from construction machinery, the proportions of halogenated hydrocarbons and alkanes emitted from aircraft were significantly higher, as were those of propionaldehyde and acetone. OVOCs were still the dominant VOC species in aircraft emissions, and their calculated ozone formation potential (OFP) was much higher than that of other VOC species at all stages of aircraft operations. Acetone, propionaldehyde, formaldehyde, acetaldehyde, and ethylene were the greatest contributors to ozone production. This study comprehensively measured the distribution characteristics of VOCs, and its results will aid in the construction of a source profile inventory of VOCs emitted from aircraft sources in real atmospheric environments.

## 1. Introduction

Volatile organic compounds (VOCs) play important roles in air quality and human health. VOCs are the precursors of O_3_ production through photochemical reactions [1], and researchers have paid increasing attention to their sources and oxidation during O_3_ pollution, especially to oxygenated volatile organic compounds (OVOCs), which are more reactive during O_3_ production [2,3,4].

The rapid economic development of China has facilitated the growth of the civil aviation industry, which is critical for long-distance travel and logistical transport [5,6]. For more than a decade, civil aviation traffic volumes have continuously increased, with an annual growth rate of 4.2% [7]. China’s civil aviation has become the second-largest air transportation system worldwide [8], with airport emissions demonstrating an increasing impact on air quality in airport regions in recent years. Studies have shown that landing and take-off (LTO) operations can release large amounts of pollutants, such as NO_x_, VOCs, fine particulate matter (PM_2.5_), CO, and SO_2_ [9]. Airport emission sources also include ground handling equipment, motor vehicles, and catering, among other emission sources [10]. Airport emissions have demonstrated regionality and centralization [11]. Large civil airports with high air passenger traffic volumes have a high impact on the surrounding air quality [12], and the resulting spatiotemporal air pollution distribution characteristics change with airport operations [13]. Currently, pollutants emitted by airports, especially VOCs, significantly contribute to urban pollution, severely threatening the ecological environment of cities and residents’ health [14]. However, few studies have been conducted on VOCs emitted by airports.

Two study methods, namely, the calculation of the emission inventory and field measurements of emissions, have mainly been employed in studies on airport VOCs. Mazaheri et al. (2011) estimated the VOC emission inventory of Brisbane Airport [15]; Winther et al. (2015) calculated this inventory for Copenhagen International Airport (Denmark) [16], and Yang et al. (2018a) calculated a detailed VOC emission inventory of Beijing Capital International Airport using the WRF-CMAQ model to perform a four-season simulation and to estimate the airport’s impact on the surrounding region [17]. Studies using field measurements have gradually increased in recent years. Gas chromatography (GC) was adopted at Zurich Airport to measure 56 types of VOCs emitted by aircraft in different operating phases [18]. Gas chromatography–mass spectrometry (GC-MS) was used at New York and New Jersey Teterboro airports to measure the impact of benzene, toluene, ethylbenzene, and xylene (BTEX) compounds emitted by aircraft on air quality in surrounding communities [19]. GC-MS with flame ionization detection was employed at Beirut Rafic Hariri International Airport to measure 48 types of VOCs emitted by aircraft under different operating states [20]. Tunable infrared laser differential absorption spectroscopy and proton-transfer-reaction mass spectrometry were used at Auckland Airport to measure the emission indices of five specific hydrocarbons, namely, formaldehyde, acetaldehyde, ethylene, propylene, and benzene [21]. In addition, GC-MS with flame ionization detection (GC-MS/FID) was adopted at Taipei International Airport to measure the concentrations of 22 types of VOCs [22]. At Beijing Capital International Airport, GC-MS/FID was used to measure 53 types of aircraft VOCs [23], while at Beijing Shahe Airport, proton-transfer-reaction time-of-flight mass spectrometry (PTR-TOF-MS) was employed to measure 16 types of VOCs [24]. In addition, the GC method was used to measure seven types of VOCs at Shanghai Hongqiao Airport, namely, 1,3-butadiene, benzene, toluene, ethylbenzene, styrene, o-xylene, and m/p-xylene [25].

So far, few studies have been conducted on the characteristics of airport emissions. Even fewer have investigated on-site emissions from aircraft during different operating stages of the LTO cycle. Additionally, the number of measured VOC types remains insufficient to construct relatively complete VOC emission profiles. A research gap is especially noticeable for OVOCs emitted from airports, mainly due to challenges and difficulties related to accessing sampling areas, the safety of sampling staff when conducting measurements, and the complexities of sampling environments, such as emissions from vehicles.

Between 2018 and 2020, passenger throughput and freight volume at Shenzhen Bao’an International Airport were among the top five airports in China, ranking third in 2020 and second in Guangdong Province (only below Guangzhou Baiyun International Airport). Zhu et al. (2023) determined that airport emissions sources were the main VOC source in Shenzhen, and the contribution rate of total VOC species concentration was 19% [26]. However, the construction of a source profile is still needed for results based on sampling in an atmospheric environment and positive matrix factorization (PMF) model calculations. Thus, Shenzhen Bao’an International Airport was selected as the research object for this study. An offline monitoring method was adopted to measure aircraft emissions on site under different operating states. The measured values of selected OVOCs and other VOCs were obtained to analyze the emission characteristics of airport pollutants, which were then used to construct their source profiles. The results of this study offer important base data for characterizing the emission characteristics of aircraft at each operating phase and can help optimize the emission inventories of VOCs at airports as well as provide an important scientific basis for the evaluation of airport emissions’ impact on air quality.

## 2. Methods

### 2.1. Sample Collection

Shenzhen Bao’an International Airport (International Air Transport Association code: SZX) is located in the Baoan District of Shenzhen, Guangdong Province (geographical coordinates: 113°49° E and 22°36° N), on the east bank of the Pearl River Estuary, 32 km away from downtown Shenzhen. During the sampling period, vehicles within the airport near the sampling sites on both sides of the runway were all electric-drive vehicles, which largely eliminated the impact of airport vehicle emissions when compared with other studies. Jet A-1 fuel, which is utilized worldwide, was used to fuel aircraft at Shenzhen airport and was produced according to international standards (ASTM D1655) https://www.astm.org/d1655-22.html, accessed on 3 March 2024.

**Figure 1:**
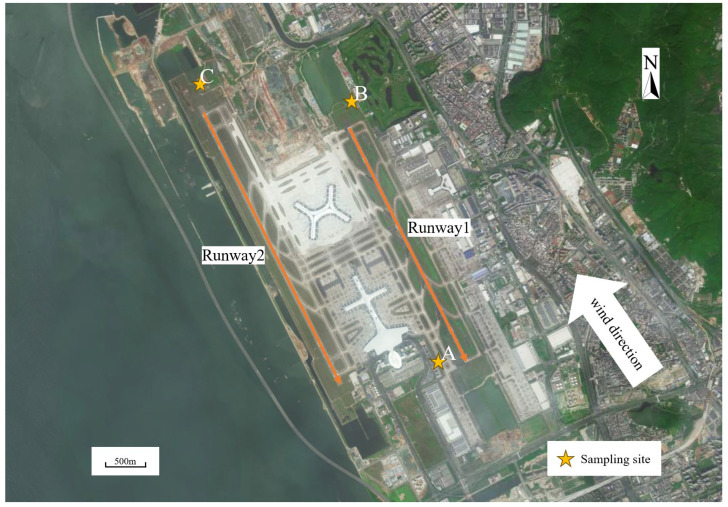
Sampling site location: the pollutants emitted by aircraft during take-off, taxiing, and approach operations were collected at points A, B, and C, respectively.

Sampling was conducted between November 9th and 21st, 2023, at three sites near the runway of Shenzhen International Airport, as shown in Figure 1. No rainfall occurred during the sampling period. Points A and B were located at the south and north ends of runway 1, respectively, while point C was located at the north end of runway 2. The pollutants emitted by aircraft during take-off, taxiing, and approach operations were collected at points A, B, and C, respectively. All points were located outside the security wire fence, about 200 m away from the runway. Wind direction was also verified to ensure that the aircraft exhaust gases could reach the sampling points, and a distinctive fuel smell could be noticed when an aircraft passed.

SilcoCan (Hangzhou Tianjing Testing Technology Co., Ltd., HHJ6000, 6 L, Hangzhou, China) was used to collect VOC samples. As shown in Table 1, the first 10 samples were collected both during taxiing and take-off (two samples per aircraft), and the last 10 were only collected during the landing phase (one per aircraft); thus, a total of 30 samples were collected. During sampling, the models and airline identification numbers of the aircraft were obtained from the air traffic activity data provided by Shenzhen Bao’an International Airport. Among these, the airline identification numbers of 10 aircraft corresponding to taxiing and take-off were identical, as shown in Table 1. Sep-Pak DNPH (dinitrophenylhydrazine)-Silica Plus short cartridges (Waters, WAT037500) were used for a 30-min short sampling of OVOCs during airport peak hours, with 10 collections performed for each operating state, for a total of 30 samples.

### 2.2. Analysis of VOCs and OVOCs

Samples collected using SilcoCan were analyzed with online adsorption concentration sampling and GC-MS systems (AC-GCMS 1000, Guangzhou Hexin Co., Ltd., Guangzhou, China) in the laboratory. The target species of VOCs consisted of 117 compounds, namely, photochemical assessment monitoring stations (PAMS) for ozone precursors (57 species), VOCs in the toxic organics (TOs)-15 standard (47 species), and aldehydes and ketones (13 species). The detailed analysis methodology was described by Jiang et al. (2023) [27].

Samples collected using Sep-Pak DNPH short cartridges were reacted through strong acid catalysis. DNPH was coated on silica gel to form stable colored hydrazone derivatives. Before measurement, pre-treatment was needed, during which the cartridges were eluted with acetonitrile (high-performance liquid chromatography (HPLC)-grade), followed by a 10-fold concentration of the eluate. The pre-treated samples were loaded into the HPLC system (LC-2000, Guangzhou Hexin Co., Ltd.) to detect 11 types of VOCs in the collected samples, i.e., formaldehyde, glyoxal, cyclohexanone, isovaleraldehyde, p-tolualdehyde, methylglyoxal, 2,5-dimethylbenzaldehyde, heptanal, octanal, nonanal, and decanal. The obtained retention times were used for qualitative identification, while the peak areas were used for qualification. The OVOCs described in the Section 3 consisted of a combination of OVOCs from the samples collected using SilcoCan and DNPH analysis. The complete species obtained using the two analysis methods are detailed in Table S1 (Supplementary Data).

### 2.3. Calculation of Ozone Formation Potential

Ozone formation potential (OFP) was used to assess the maximum contribution of different VOC species to O_3_ formation under optimal reaction conditions. OFP is the product of the environmental concentration of one VOC species and its maximum incremental reactivity (MIR) according to the following equation:

OFP_i_ = MIR_i_ × C_i_,

where C_i_ is the mass concentration of a VOC species emitted by an aircraft, and MIR_i_ denotes the ozone generation coefficient in the maximum ozone incremental reaction in the maximum ozone incremental reaction. The MIR coefficient reported by Venecek et al. (2018) was used in this study [28]. Specific MIR coefficients are shown in Table S2. Species without MIR values were excluded from OFP calculations.

## 3. Results and Discussion

### 3.1. Characteristics of VOCs Emitted at Different Flight States

According to the regulations imposed by the International Civil Aviation Organization (ICAO), the thrust levels of aircraft during the taxiing (idle), approach, and take-off stages must be 7%, 30%, and 100% of the rated thrust, respectively [29], with different thrust levels reflecting differences in aircraft engine temperature and fuel combustion level. The total mass concentrations of VOCs measured in this study during taxiing, approach, and take-off were 154.67, 192.60, and 132.24 μg/m^3^, respectively. The lowest and highest VOC mass concentrations were measured during take-off and approach, respectively. These distribution patterns of VOCs during take-off and approach phases were similar to the measurements made by Mokalled et al. (2019) using GC-MS [20]. The VOC concentrations measured during the taxiing phase were within the reported range of 140–400 μg/m^3^ measured by Schürmann et al. (2007) [18].

**Figure 2:**
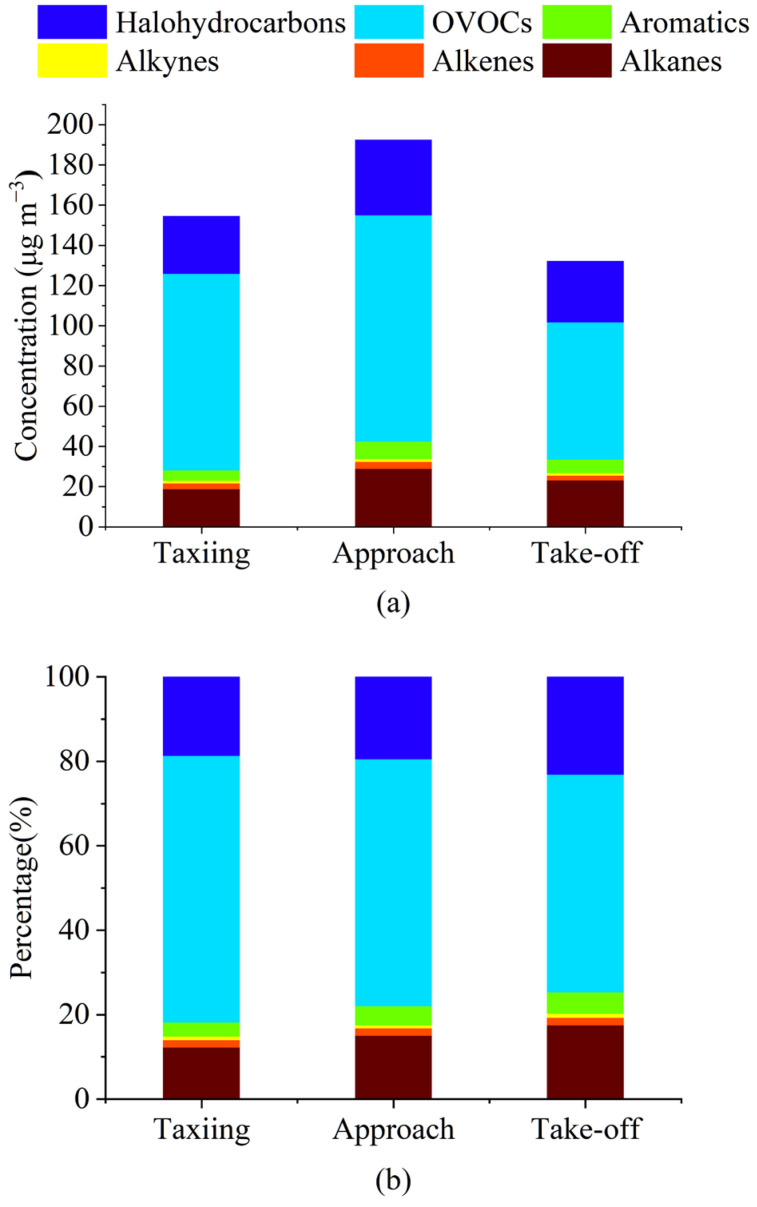
Concentration composition and proportion of the airport source VOCs measured under three operating conditions: (**a**) VOC concentrations; and (**b**) weight %.

As shown in Figure 2, OVOCs were the main components of VOC emissions in the taxiing, approach, and take-off states, and their mass concentration proportions were 63.17%, 58.44%, and 51.60%, respectively. The second component was halogenated hydrocarbons, with mass concentration proportions of 18.73%, 19.59%, and 23.18%, respectively. A study by Spicer et al. (2009) found that halogenated hydrocarbons emitted from aircraft engines operating at minimum power were lower than those emitted at maximum power [30]. The proportion of OVOCs decreased with increasing aircraft thrust, while the proportion of halogenated hydrocarbons increased. During taxiing, approach, and take-off, the thrust of the engine sequentially increases, and with increasing thrust, the temperature inside the aircraft engine also increases [20]. A previous study found that the concentration of carbonyl compounds in OVOCs was greatly affected by temperature, and with increasing temperature, the concentration of carbonyl compounds correspondingly decreased [31,32]. We measured more types of OVOCs than previous studies on airport emissions (30 species of 33 target OVOCs were detected using the SilcoCan and DNPH sampling cartridges). This was likely the main reason why the mass concentration proportions of OVOCs were significantly higher than the results obtained in previous studies.

The mass concentration percentages of both alkanes and aromatic hydrocarbons increased with an increase in aircraft engine thrust, which corresponded to the results obtained in studies by Anderson et al. (2006) [33], Lelievre (2009) [34], and Mokalled et al. (2019) [20]. In this study, the mass concentration proportions of alkanes during taxiing, approach, and take-off were 12.15%, 14.96%, and 17.42%, respectively, while the respective mass concentration proportions of aromatic hydrocarbons produced during taxiing, approach, and take-off were 3.30%, 4.53%, and 5.07%. The contributions of alkenes and alkynes toward aircraft emissions were relatively small, and they were less affected by changes in aircraft engine thrust. Their mass concentration proportions showed almost no change during taxiing, approach, and take-off; those of alkenes during taxiing, approach, and take-off were 1.79%, 1.80%, and 1.83%, while those of alkynes were 0.86%, 0.69%, and 0.90%, respectively.

### 3.2. Distribution Characteristics of VOCs Emitted from Airport Sources

#### 3.2.1. Composition Characteristics of the Main Chemical Components of Each Type of VOC

**Figure 3:**
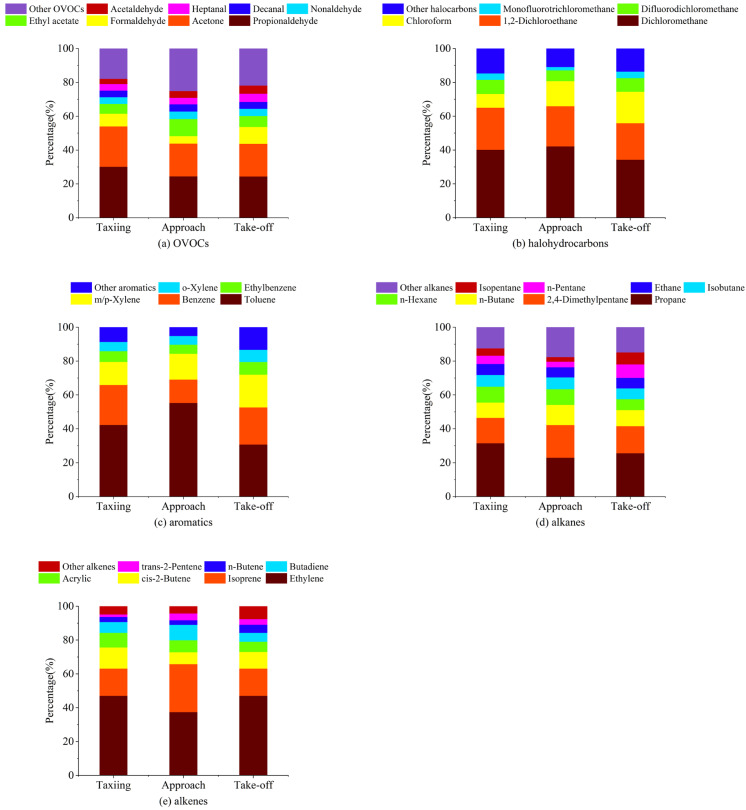
Distribution characteristics of (**a**) OVOCs, (**b**) halogenated hydrocarbons, (**c**) aromatics, (**d**) alkanes, and (**e**) alkenes obtained using GC-MS/FID and HPLC.

The component proportions of each type of VOC during different flight states are shown in Figure 3. Among OVOCs, acetone and propionaldehyde demonstrated the highest proportions, which was similar to the results obtained by Mokalled et al. (2019) at Beirut Rafic Hariri International Airport [20]. In our study, the total proportions of acetone and propionaldehyde during taxiing, approach, and take-off were 53.92%, 43.77%, and 43.67%, respectively, and their total proportions decreased with increasing aircraft engine thrust. This phenomenon was also found in the results obtained by Spicer et al. (1994) [35]. Formaldehyde was collected using DNPH-coated silica extraction cartridges, and its concentration was greatly affected by dilution; however, it showed relatively high mass concentration proportions under the three aircraft operating states, with values of 7.53%, 4.42%, and 9.93%, respectively. The measurement results obtained by Beyersdorf et al. (2012) found that formaldehyde was the main carbonyl compound emitted [36], and a relatively high mass concentration proportion of ethyl acetate (10.10%) was found in aircraft during the approach. Since a high concentration of ethyl acetate has been found in airplane cabins [37], it has been speculated that ethyl acetate might be related to the cabin air exchange system. Acetaldehyde, a common OVOC species, is a photochemical oxidant with high reactivity [38]. In the measurement results obtained in this study, its mass concentration proportion among total OVOCs was insignificant and amounted to 3.04%, 4.04%, and 4.84% during taxiing, approach, and take-off, respectively. Nonanal, decanal, and heptanal were not observed in other airport emissions studies. However, in this study, we found that their mass concentration proportions during each operating state all exceeded 3%.

We observed a high mass concentration of halogenated hydrocarbons, among which dichloromethane was the most abundant. Dichloromethane is typically found in paints and coatings and is a chlorine-containing halocarbon produced primarily from anthropogenic sources [39]. During taxiing, take-off, and approach, the mass concentration proportions of dichloromethane among the total emitted halogenated hydrocarbons were 40.11%, 34.21%, and 42.06%, respectively. Chlorinated organic compounds observed by MacGregor et al. (2008) in aircraft cabins and bleed air also included dichloromethane [40]. The second highest contribution was that of 1,2-dichloroethane; its mass concentration proportions during taxiing, take-off, and approach were 24.89%, 23.79%, and 21.62%, respectively, and decreased with increasing engine thrust. Chloroform and difluorodichloromethane also demonstrated relatively high mass concentration proportions, with chloroform increasing with increasing engine thrust. A study by Spicer et al. (2009) found that dichloromethane, chloroform, and difluorodichloromethane were emitted by aircraft turbine engines under various thrust conditions [30]. In a study by Yin et al. (2022), the concentration of chloroform among halogenated hydrocarbons was more significant in aircraft nacelles. Its source was apparently related to the use of detergents in cleaning activities, and trichloromethane and 1,2-dichloroethane are volatile organic compounds of concern [41]. Air induction in an aircraft cabin is mainly carried out by the engine. The ground engine’s power is the lowest during taxiing, which is when the gas exchange efficiency of the cabin is the lowest in relative terms. With increasing engine power, the gas exchange efficiency of the cabin increases, and more chloroform will be emitted. Chloroform oxidizes into highly toxic phosgene when exposed to air in the presence of light, and its health impact and environmental toxicity cannot be ignored [42]. Trichlorofluoromethane was also detected during measurement, exhibiting mass concentration proportions of 3.82%, 1.95%, and 3.88% during taxiing, approach, and take-off, respectively. Trichlorofluoromethane is typically used as a refrigerant; it has certain health hazards and can damage the ozone layer.

Toluene and benzene were found to be the dominant aromatic hydrocarbons by mass emitted by aircraft at each operating state. The mass concentration proportions of toluene among the aromatic hydrocarbons emitted during taxiing, approach, and take-off were 42.21%, 55.16%, and 30.57%, respectively, while those of benzene were 23.59%, 13.87%, and 21.97%, respectively. The mass concentration proportions of m- and p-xylene were also relatively high: 13.69%; 15.19%; and 19.38%, respectively. Lai et al. (2013) found high mass concentrations of toluene, benzene, and m- and p-xylene at the parking apron of Taiwan Taipei International Airport and observed that ethylbenzene and o-xylene had significantly higher mass concentrations than other aromatic hydrocarbons [22]. Thus, the main form of aromatic hydrocarbons emitted by aircraft was benzene series (BTEX) compounds. Beyersdorf et al. (2012) found that ethylbenzene and o-xylene were important components of aromatic hydrocarbons emitted by aircraft engines [36].

Propane was the most abundant among alkanes. Its mass concentration proportions during taxiing, approach, and take-off were 31.41%, 22.90%, and 25.54%, respectively. The second highest proportion was that of 2,4-dimethylpentane; its mass concentration proportions were 14.96%, 19.24%, and 15.93%, respectively. The concentration of this species measured near the runway of Beijing Capital International Airport was found to be significantly different from the background point concentration [23]. Other alkanes with relatively high contents were n-butane, n-hexane, isobutane, ethane, n-pentane, and isopentane; their mass concentration proportions at each operating state all exceeded 3% except for the value of isopentane during the approach, which was 2.68%. The concentration of ethane was not high, similar to the results obtained by Mokalled et al. (2019) [20].

As for alkenes, ethylene and isoprene were the dominant species. The mass concentration proportions of ethylene among alkenes emitted during taxiing, approach, and take-off were 46.98%, 37.37%, and 46.97%, respectively, and the corresponding values of isoprene were 16.13%, 28.35%, and 16.10%. Compared with the other two operating states, the proportion of ethylene during the approach was relatively low, while that of isoprene was relatively high. Ethylene was found to be the main alkene emitted by aircraft [20,43], while isoprene was also found to be emitted in previous airport studies [18,22]. The mass concentration proportions of cis-2-butene, propylene, and butadiene were similar and were all higher than 5% at each operating state. The mass concentration proportions of n-butene and trans-2-pentene were relatively low, regardless of operating state, and were around 3%. Among these, cis-2-butene, n-butene (1-butene), and trans-2-pentene (in descending order by concentration) were observed in the measurement results at Santos Dumont Airport [44]. Propylene is a common alkene; its mass concentration proportions observed during taxiing, approach, and take-off in this study were 8.61%, 7.08%, and 5.99%, respectively, and decreased with increasing aircraft engine thrust. It is worth noting that high concentrations of propylene were found in previous studies on aircraft engine emissions [36].

#### 3.2.2. Comparison with Other Mobile Sources

**Figure 4:**
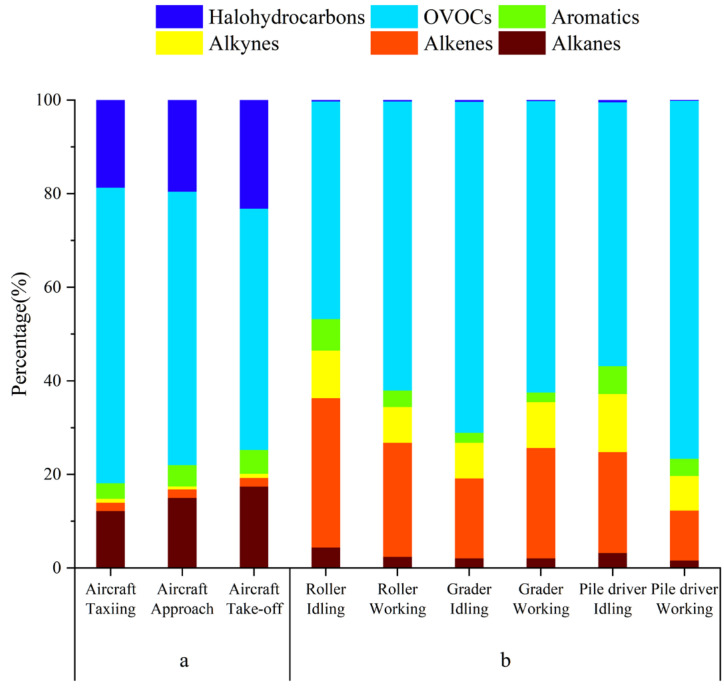
Proportion of VOCs from (**a**) airport sources and (**b**) construction site emission sources. Data for construction machinery sources are from [45].

To differentiate airport sources and the surrounding construction site, we compared our results with the component distribution characteristics of the construction machinery [45], as shown in Figure 4. The proportions of halogenated hydrocarbons and alkanes emitted by aircraft were distinctively high and were in the ranges of 18.73–23.18% and 12.15–17.42%, respectively. However, the proportions of alkenes and alkynes from the construction machinery were substantially higher than those from aircraft emissions: alkenes accounted for 10.7–31.9% of the construction machinery emissions. The proportions of OVOCs from aircraft emissions and construction machinery emissions were 51.60–63.17% (Table 2) and 46.9–76.5%, respectively. OVOCs were the main component of emitted VOCs in both emission sources. The proportion of OVOCs in VOCs emitted by gasoline-powered vehicles was the lowest (13%); therefore, its value could be used to differentiate aircraft and gasoline-powered vehicle exhaust sources. However, OVOCs were also the main components of VOCs emitted from diesel-powered cars and trucks; their proportions were 49% and 42.7–69.2%, respectively. Therefore, if aircraft and construction site emission sources are to be differentiated by OVOCs, their species composition characteristics need to be analyzed. Among the OVOCs emitted by aircraft, propionaldehyde and acetone had the highest proportions, with values of 24.31–30.01% and 19.37–23.91%, respectively. For construction machinery, the highest proportions were found for acetaldehyde and formaldehyde, with respective values of 25.09–30.15% and 12.94–23.33%. Acetaldehyde and formaldehyde are also the main components of OVOCs emitted from diesel engines, including diesel-powered trucks [46,47,48,49].

### 3.3. Concentration Characteristics of the Main VOCs Emitted by Aircraft during Different Operating States

Table 3 presents the top 10 VOC species emitted by aircraft in each phase and their average mass concentrations. In the taxiing phase, the volatile organic compounds with the highest mass concentrations were propionaldehyde (18.95%), acetone (15.10%), methylene chloride (7.51%), and formaldehyde (4.75%), while during approach, the principal pollutants were propionaldehyde (14.23%), acetone (11.34%), methylene chloride (8.24%), and ethyl acetate (5.90%). During take-off, propionaldehyde (12.54%), acetone (9.99%), methylene chloride (7.93%), and formaldehyde (5.12%) predominated. Among these, during the three operating states, propionaldehyde, acetone, and methylene chloride were the top three compounds in terms of concentration. At least half of the top 10 species in aircraft emissions in terms of mass concentration in all operating conditions were OVOCs. OVOC species also accounted for the highest proportion of compounds emitted by aircraft during each operating state. It should be noted that some semi-volatile and low-volatile organics (such as aromatics with three and four rings) are distributed in gas and particle phases [50,51], and the measurement of these species in the gas phase cannot depict their comprehensive emission characteristics from the aircrafts. In this study, the low concentration of aromatics only demonstrated their performance in the gas phase, which did not represent the total amount emitted from the aircrafts. A part of aromatics could be directly emitted in the particle phase together with soot and ultrafine particles, which have been found in biomass burning and vehicle exhaust measurements [52,53,54].

### 3.4. Source Profile Characteristics of Aircraft Emissions

**Figure 5:**
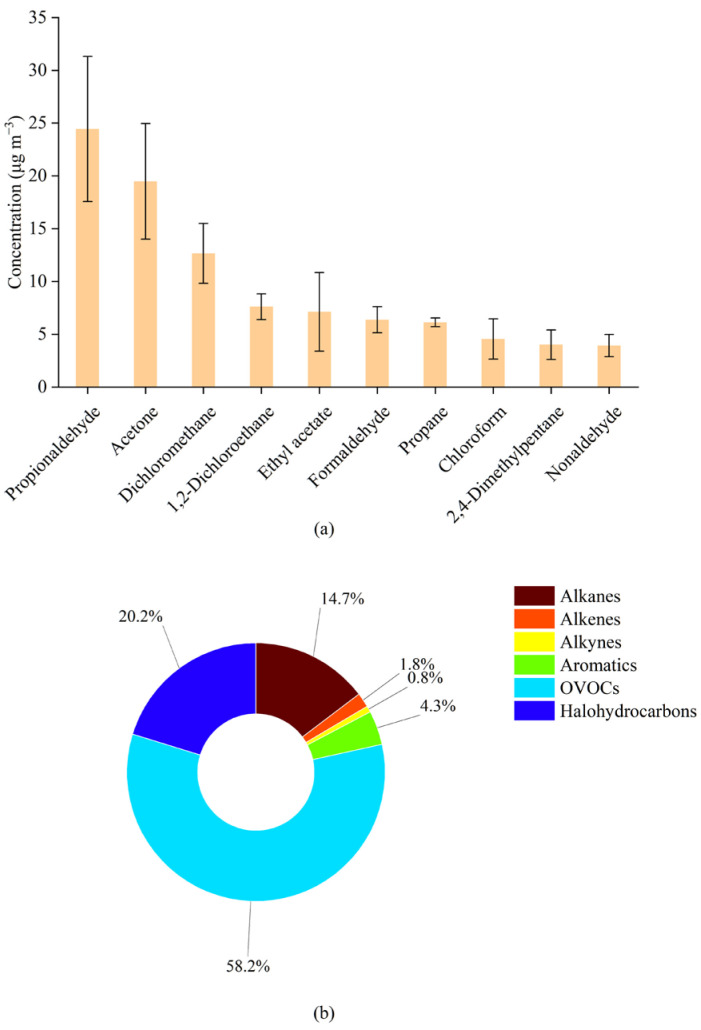
(**a**) Top 10 species emitted by aircraft by VOC source profile concentration. (**b**) Distribution of total VOCs in the source profile by compound category (unit: weight %).

In this study, the source profiles of aircraft emissions at Shenzhen Bao’an International Airport were obtained by calculating the mean concentration values of the samples collected at each sampling point. These source profiles are provided in Table S3 (Supplementary Data), and the top 10 VOCs species emitted by aircrafts well as the distribution of total VOCs in the source profile are presented in Figure 5.

### 3.5. Comparison of OFP Values in Different Operating States

**Figure 6:**
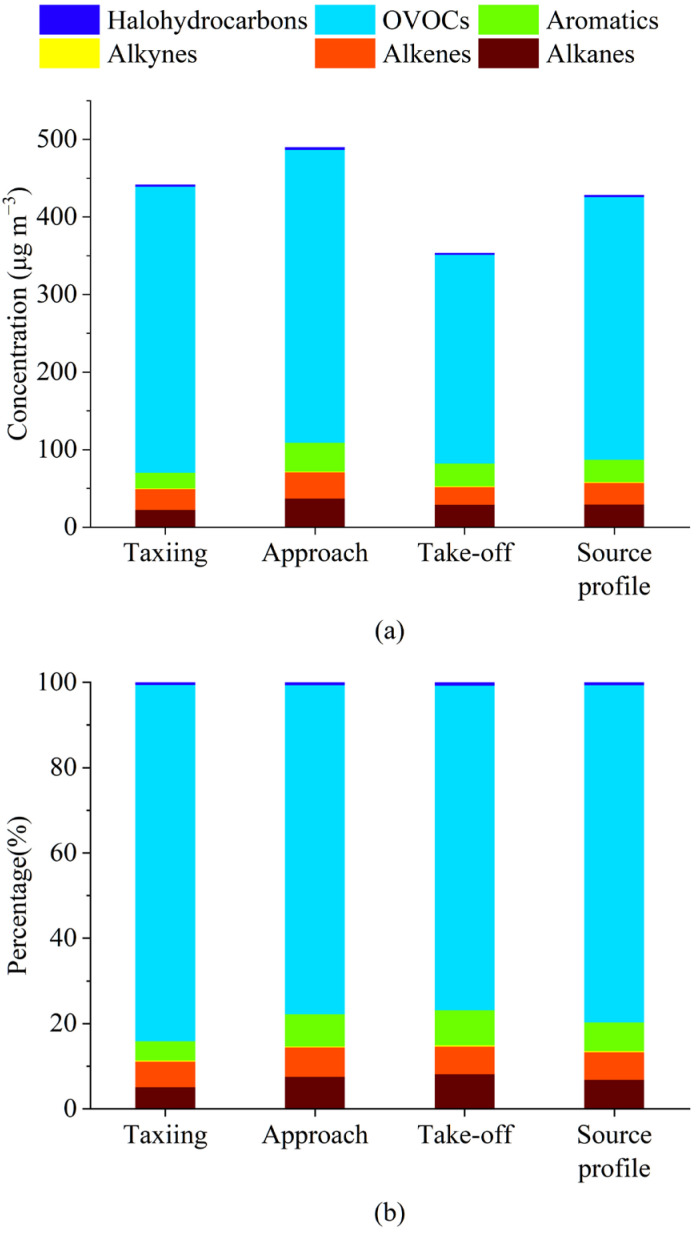
Total OFP distribution by compound class for aircraft emissions at different operating states: (**a**) mass concentrations; and (**b**) weight %.

The MIR of VOC species was used to calculate their OFP values [28,55].As shown in Figure 6a, the highest OFP was found during the approach, followed by the taxiing phase, and was the lowest during take-off. This had a certain correlation with the differences in mass concentration corresponding to the three operating states. Meanwhile, some important OVOCs, such as propionaldehyde, formaldehyde, and acetaldehyde, demonstrated relatively high MIR. The differences in the OFP contributions of alkanes, aromatics, and halogenated hydrocarbons during the three operating states showed the same patterns as their mass concentration proportions, which all increased with increasing engine thrust. Notably, although they had a low mass concentration proportion, alkenes significantly contributed toward OFP generation; this phenomenon could be attributed to the fact that alkenes generally demonstrated high MIR [45].

In the source profile, OVOCs were the predominant contributor to OFP, and their proportion was 79.03%. Therefore, in order to control the ozone generation caused by aircraft, attention should be paid to OVOCs.

Table 4 presents the top 10 components emitted by aircraft under various operating states in terms of OFP value. It is evident that the proportion of OVOCs exceeded 50%. Among them, except for propionaldehyde, which had a high MIR and high mass concentration, the high OFPs of other species were related to their corresponding MIR values. For emissions during taxiing, approach, and take-off, the top 10 OFP species accounted for 71.84–79.45% of total OFP. Therefore, to investigate the impact of VOCs emitted by aircraft on ozone production, these species should primarily be considered. Among them, during the approach, the contribution of OVOCs to OFP was dominant, and only ethylene and toluene were not OVOC species. Ethylene and toluene were also found among the top 10 species in OFP during approach and take-off, suggesting that ethylene and toluene were notable species. During the approach, the OFP contributions of toluene and 2,4-dimethylpentane also increased to some extent. Additionally, isoprene was among the top 10 species contributing to OFP, which was related to its high mass concentration during the approach period.

## 4. Conclusions

In this study, field measurements of VOCs emitted by aircraft were conducted at Shenzhen Bao’an International Airport during different LTO cycle operating states. This study provided a more complete composition of VOCs (especially OVOCs) emitted by aircraft than previous airport studies. For aircraft emissions on the ground in various LTO cycle operating states, OVOCs were the main species, and their mass concentration proportion exceeded 50%. Among these, propionaldehyde and acetone were the primary pollutants, followed by halogenated hydrocarbons, which showed significant differences from other mobile sources in terms of composition. Overall, the measured mass concentration of total pollutants during the take-off phase was the lowest, and the value during the approach phase was the highest. The contributing proportion of OVOCs was the highest during taxiing, and with increasing aircraft engine thrust (from taxiing to take-off), its proportion gradually decreased. At the same time, the mass concentration proportions of halogenated hydrocarbons, alkanes, and aromatic hydrocarbons gradually increased. The above findings are associated with the fact that engine temperature increases along with increasing thrust. Compared with other mobile sources, the distribution characteristics of components emitted by aircraft were dramatically different. The proportions of halogenated hydrocarbons and alkanes emitted by aircraft were much higher than those emitted by construction machinery. The proportion of OVOCs in aircraft emissions was significantly higher than in those from gasoline-powered vehicles, and the proportions of propionaldehyde and acetone emitted by aircraft were noticeably higher than those emitted by other mobile sources. OVOCs were found to contribute to the OFP at each of the operating states, and their contribution was related to their concentration proportions. These results suggest that OVOCs should be paid attention to in future studies on the sources of airport ozone production. This study also estimated the source profiles of on-site aircraft emissions. Our results will aid subsequent studies on airport pollutants.

## Figures and Tables

**Table 1:** Airline identification numbers and models of 10 aircraft corresponding to taxiing, approach, and take-off.

Operating Status	Flight Number	Aircraft Type
Taxiing and take-off	CZ3587	Airbus A330-343
Taxiing and take-off	CZ5755	Airbus A320-232
Taxiing and take-off	ZH9429	Boeing 737-87L
Taxiing and take-off	CZ6310	Airbus A320-251N
Taxiing and take-off	MU5348	Airbus A350-941
Taxiing and take-off	MU5244	Airbus A321-211
Taxiing and take-off	ZH9887	Boeing 737-87L
Taxiing and take-off	CZ3225	Airbus A330-323
Taxiing and take-off	CA1734	Airbus A330-343
Taxiing and take-off	TV9902	Airbus A330-243
Approach	9C8775	Airbus A321-253NX
Approach	CA1303	Airbus A330-243
Approach	MU2887	Airbus A320-214
Approach	DZ6242	Boeing 737-8HX
Approach	ZH9210	Airbus A320-271N
Approach	ZH9602	Airbus A319-133
Approach	HU7358	Boeing 787-9
Approach	ZH9104	Airbus A320-271N
Approach	CZ6706	Boeing 737-81B
Approach	CA4311	Airbus A321-251NX

**Table 2:** Comparison of the proportion of OVOCs in VOCs emitted by aircraft with the proportion of OVOCs from other mobile sources reported in the literature.

Emission Source	Analytical Method	OVOCs as a Percentage of Total VOCs	
Aircraft	GC-MS/FID and HPLC	51.60–63.17%	This work
Roller	GC-MS/FID and HPLC	60.10%	Wang et al. (2020) [45]
Grader		63.00%	Wang et al. (2020) [45]
Pile driver		74.80%	Wang et al. (2020) [45]
Gasoline car	GC-MS/FID and PTR-ToF-MS	13%	Wang et al. (2022) [46]
Diesel car		49%	Wang et al. (2022) [46]
Liquefied petroleum gas vehicles		58%	Wang et al. (2022) [46]
Diesel truck	GC-MS/FID and HPLC	42.7–69.2%	Yao et al. (2015) [47]

**Table 3:** Mass concentrations of the top 10 VOC species emitted by aircraft in each operating state.

Operating Stage	Species	Concentration ± Standard Deviation (Unit: μg/m^3^)
Taxiing	Propionaldehyde	29.32 ± 16.57
	Acetone	23.36 ± 13.2
	Dichloromethane	11.62 ± 3.13
	Formaldehyde	7.35 ± 2.53
	1,2-Dichloroethane	7.21 ± 2.59
	Propane	5.9 ± 2.29
	Ethyl	5.61 ± 1.69
	Nonaldehyde	3.89 ± 1.23
	Decanal	3.83 ± 0.84
	Heptanal	3.79 ± 0.73
Approach	Propionaldehyde	27.42 ± 2.66
	Acetone	21.85 ± 2.12
	Dichloromethane	15.87 ± 3.35
	Ethyl	11.37 ± 2.37
	1,2-Dichloroethane	8.98 ± 3.05
	Propane	6.6 ± 1.8
	Chloroform	5.59 ± 4.03
	2,4-Dimethylpentane	5.54 ± 3.13
	2-Butanone	5.13 ± 1.11
	Nonaldehyde	5 ± 1.51
Take-off	Propionaldehyde	16.58 ± 1.87
	Acetone	13.22 ± 1.49
	Dichloromethane	10.49 ± 1.46
	Formaldehyde	6.77 ± 3.62
	1,2-Dichloroethane	6.63 ± 2.76
	Propane	5.88 ± 1.05
	Chloroform	5.71 ± 2.99
	Ethyl	4.4 ± 1.07
	2,4-Dimethylpentane	3.67 ± 0.93
	Heptanal	3.31 ± 0.52

**Table 4:** Distribution of the top 10 OFP species emitted by aircraft at each operating state.

Operating Stage	Species	Concentration ± Standard Deviation (μg/m^3^)
Taxiing	Propionaldehyde	198.77 ± 112.33
	Formaldehyde	52.64 ± 18.12
	Acetaldehyde	18.04 ± 1.49
	Glyoxal	17.57 ± 5.98
	Methylglyoxal	16.63
	Heptanal	13.44 ± 2.6
	Ethylene	11.23 ± 2.53
	Toluene	8.66 ± 1.74
	Acetone	8.01 ± 4.53
	Hexanal	5.88 ± 5.17
Approach	Propionaldehyde	185.88 ± 18.04
	Formaldehyde	35.65 ± 18.08
	Acetaldehyde	27.62 ± 2.84
	Methylglyoxal	24.3 ± 0.18
	Toluene	19.32 ± 7.17
	Heptanal	15.19 ± 4.41
	Glyoxal	13.41 ± 3.64
	Ethylene	11.19 ± 5.41
	2,4-Dimethylpentane	9.76 ± 5.51
	Isoprene	9.54 ± 1.89
Take-off	Propionaldehyde	112.44 ± 12.71
	Formaldehyde	48.5 ± 25.91
	Methylglyoxal	20.08 ± 6.66
	Acetaldehyde	20.03 ± 2.27
	Glyoxal	17.26 ± 3.9
	Heptanal	11.74 ± 1.86
	Ethylene	9.79 ± 4.66
	m/p-Xylene	9.09 ± 7
	Toluene	8.24 ± 3.68
	2,4-Dimethylpentane	6.46 ± 1.64

## Data Availability

The data presented in this study are available on request from the corresponding author Mei Li due to professional guidance for SPAMS data.

## Supplementary Materials

The following supporting information can be downloaded at: https://www.mdpi.com/article/10.3390/toxics12040243/s1, Table S1: All species measured by GC-MS/FID and HPLC; Table S2: MIR values corresponding to each species; Table S3: Source profile of aircraft emissions.
