# Supplementary material for: Characteristics of Volatile Organic Compounds Emitted from Airport Sources and Their Effects on Ozone Production
Source: Toxics. 2024 Mar 26;12(4):243. doi: 10.3390/toxics12040243 (PMC11053784; doi:10.3390/toxics12040243)
Supplement: Supplementary file 1 [file toxics-12-00243-s001.zip › toxics-2925752-supplementary.pdf]

Appendix A. Supplementary data for "Characteristics of volatile organic compounds emitted from airport sources and their effects on ozone"

Mubai Chen[1], Shiping Li[2], Long Yun[2], Yongjiang Xu[1], Daiwei Chen[1], Chuxiong Lin[2], Zhicheng Qiu[2], Yinong You[1], Ming Liu[3], Zhenrong Luo[3], Liyun Zhang[3], Chunlei Cheng[1,4]*, Mei Li[1,4]*

[1]Institute of Mass Spectrometry and Atmospheric Environment, Guangdong Provincial Engineering Research Center for Online Source Apportionment System of Air Pollution, Jinan University, Guangzhou 510632, China

[2]Shenzhen Ecological and Environmental Monitoring Center of Guangdong Province, Shenzhen 518049, China

[3]Guangzhou Hexin Instrument Co., Ltd, Guangzhou 510530, China

[4]Guangdong-Hongkong-Macau Joint Laboratory of Collaborative Innovation for Environmental Quality, Guangzhou 510632, China

*Correspondence to: Chunlei Cheng (chengcl.vip@foxmail.com) and Mei Li (limei2007@163.com)

Tel: 86-20-85225991, Fax: 86-20-85225991

Table S1. All species measured by GC-MS/FID and HPLC.

No.	Species	No.	Species	No.	Species	No.	Species
1	Ethane	33	Carbon disulfide	65	Crotonaldehyde	97	1-Ethyl-3-methylbenzene
2	Ethylene	34	Isopropyl alcohol	66	Trichlorethylene	98	p-Ethyltoluene
3	Propane	35	Dichloromethane	67	Methylcyclohexane	99	1,3,5-Trimethylbenzene
4	Difluorodichloromethane	36	2,3-Dimethylbutane	68	1,2-Dichloropropane	100	Decane
5	Acrylic	37	2-Methylpentane	69	Valeraldehyde	101	1-Ethyl-2-methylbenzene
6	Isobutane	38	Cyclopentane	70	Methyl methacrylate	102	Benzaldehyde
7	n-Butane	39	trans-1,2-Dichloroethylene	71	1,4-Dioxane	103	1,2,4-Trimethylbenzene
8	Acetylene	40	3-Methylpentane	72	Bromodichloromethane	104	1,3-Dichlorobenzene
9	trans-2-Butene	41	Methyl tert-butyl ether	73	2,3,4-Trimethylpentane	105	p-Dichlorobenzene
10	cis-2-Butene	42	1-Hexene	74	2-Methylheptane	106	1,2,3-Trimethylbenzene
11	n-Butene	43	n-Hexane	75	trans-1,3-Dichloro-1-propene	107	Chlorotoluene
12	1,1,2,2-Tetrafluoro-1,2-dichloroethane	44	Methacrolein	76	3-Methylheptane	108	1,3-Diethylbenzene
13	Methyl chloride	45	1,1-Dichloroethane	77	4-Methyl-2-pentanone	109	o-Dichlorobenzene
14	Vinyl chloride	46	Vinyl acetate	78	Toluene	110	p-Diethylbenzene
15	Butadiene	47	2,4-Dimethylpentane	79	n-Octane	111	Undecane
16	Acetaldehyde	48	n-Butyraldehyde	80	cis-1,3-Dichloro-1-propene	112	3-Methylbenzaldehyde
17	Methyl bromide	49	Methylcyclopentane	81	1,1,2-Trichloroethane	113	Dodecane
18	Ethyl chloride	50	cis-1,2-dichloroethylene	82	Tetrachlorethylene	114	1,2,4-Trichlorobenzene
19	Isopentane	51	2-Butanone	83	2-Hexanone	115	1,1,2,3,4,4-Hexachloro-1,3-butadiene
20	Mono-fluoro-trichloromethane	52	Ethyl acetate	84	Dibromochloromethane	116	Naphthalene

No.	Species	No.	Species	No.	Species	No.	Species
21	1-Pentene	53	Chloroform	85	Hexanal	117	Formaldehyde
22	n-Pentane	54	Tetrahydrofuran	86	1,2-Dibromoethane	118	Glyoxal
23	trans-2-Pentene	55	1,1,1-Trichloroethane	87	Chlorobenzene	119	Cyclohexanone
24	Isoprene	56	2-Methylhexane	88	Ethylbenzene	120	Isovaleraldehyde
25	cis-2-Pentene	57	Cyclohexane	89	m/p-Xylene	121	p-Methylbenzaldehyde
26	Ethanol	58	2,3-Dimethylpentane	90	n-Nonane	122	Methylglyoxal (Pyruvaldehyde)
27	Acrolein	59	Carbon tetrachloride	91	o-Xylene	123	2,5-Dimethylbenzaldehyde
28	Propionaldehyde	60	3-Methylhexane	92	Styrene	124	Heptanal
29	1,1-Dichloroethylene	61	Benzene	93	Bromoform	125	Octanal
30	1,2,2-Trifluoro-1,1,2-trichloroethane	62	1,2-Dichloroethane	94	Cumene	126	Nonaldehyde
31	2,2-Dimethylbutane	63	2,2,4-Trimethylpentane	95	Symmetric tetrachloroethane	127	Decanal
32	Acetone	64	n-Heptane	96	n-Propylbenzene		

Table S2. MIR values corresponding to each species

No.	Species	Chemical groups	MIR
1	Ethane	Alkanes	0.32
2	Ethylene	Alkenes	8.64
3	Propane	Alkanes	0.56
4	Difluorodichloromethane	Halohydrocarbons	–
5	Acrylic	Alkenes	10.80
6	Isobutane	Alkanes	1.30
7	n-Butane	Alkanes	1.33
8	Acetylene	Alkynes	0.94
9	trans-2-Butene	Alkenes	12.50
10	cis-2-Butene	Alkenes	12.20
11	n-Butene	Alkenes	9.30
12	1,1,2,2-Tetrafluoro-1,2-dichloroethane	Halohydrocarbons	–
13	Methyl chloride	Halohydrocarbons	0.04
14	Vinyl Chloride	Halohydrocarbons	3.19
15	Butadiene	Alkenes	11.50
16	Acetaldehyde	OVOCs	6.07
17	Methyl bromide	Halohydrocarbons	0.02
18	Ethyl chloride	Halohydrocarbons	0.34
19	Isopentane	Alkanes	1.65
20	Monofluorotrichloromethane	Halohydrocarbons	–
21	1-Pentene	Alkenes	6.92
22	n-Pentane	Alkanes	1.56
23	trans-2-Pentene	Alkenes	9.74
24	Isoprene	Alkenes	9.71
25	cis-2-Pentene	Alkenes	9.62
26	Ethanol	OVOCs	1.79
27	Acrolein	OVOCs	6.98
28	Propionaldehyde	OVOCs	6.78
29	1,1-Dichloroethylene	Halohydrocarbons	2.08
30	1,2,2-Trifluoro-1,1,2-trichloroethane	Halohydrocarbons	–
31	2,2-Dimethylbutane	Alkanes	1.30
32	Acetone	OVOCs	0.34
33	Carbon disulfide	Organic sulfur	0.25
34	Isopropyl alcohol	OVOCs	0.64
35	Dichloromethane	Halohydrocarbons	0.05
36	2,3-Dimethylbutane	Alkanes	1.09
37	2-Methylpentane	Alkanes	1.77
38	Cyclopentane	Alkanes	2.37
39	trans-1,2-Dichloroethylene	Halohydrocarbons	1.80
40	3-Methylpentane	Alkanes	2.09
41	Methyl tert-butyl ether	OVOCs	0.79
42	1-Hexene	Alkenes	5.47
43	n-Hexane	Alkanes	1.55

No.	Species	Chemical groups	MIR
44	Methacrolein	OVOCs	5.43
45	1,1-Dichloroethane	Halohydrocarbons	0.08
46	Vinyl acetate	OVOCs	2.87
47	2,4-Dimethylpentane	Alkanes	1.76
48	n-Butyraldehyde	OVOCs	5.73
49	Methylcyclopentane	Alkanes	2.23
50	cis-1,2-dichloroethylene	Halohydrocarbons	–
51	2-Butanone	OVOCs	1.53
52	Ethyl acetate	OVOCs	0.72
53	Chloroform	Halohydrocarbons	0.03
54	Tetrahydrofuran	OVOCs	4.77
55	1,1,1-Trichloroethane	Halohydrocarbons	0.01
56	2-Methylhexane	Alkanes	1.57
57	Cyclohexane	Alkanes	1.81
58	2,3-Dimethylpentane	Alkanes	1.55
59	Carbon tetrachloride	Halohydrocarbons	–
60	3-Methylhexane	Alkanes	1.91
61	Benzene	Aromatics	0.79
62	1,2-Dichloroethane	Halohydrocarbons	0.23
63	2,2,4-Trimethylpentane	Alkanes	1.38
64	n-Heptane	Alkanes	1.37
65	Crotonaldehyde	OVOCs	8.18
66	Trichlorethylene	Halohydrocarbons	0.75
67	Methylcyclohexane	Alkanes	1.86
68	1,2-Dichloropropane	Halohydrocarbons	0.32
69	Valeraldehyde	OVOCs	4.88
70	Methyl methacrylate	OVOCs	11.70
71	1,4-Dioxane	OVOCs	2.68
72	Bromodichloromethane	Halohydrocarbons	–
73	2,3,4-Trimethylpentane	Alkanes	1.20
74	2-Methylheptane	Alkanes	1.37
75	trans-1,3-Dichloro-1-propene	Halohydrocarbons	4.73
76	3-Methylheptane	Alkanes	1.53
77	4-Methyl-2-pentanone	OVOCs	3.81
78	Toluene	Aromatics	4.02
79	n-Octane	Alkanes	1.15
80	cis-1,3-Dichloro-1-propene	Halohydrocarbons	3.66
81	1,1,2-Trichloroethane	Halohydrocarbons	0.09
82	Tetrachlorethylene	Halohydrocarbons	0.04
83	2-Hexanone	OVOCs	3.47
84	Dibromochloromethane	Halohydrocarbons	–
85	Hexanal	OVOCs	4.17
86	1,2-Dibromoethane	Halohydrocarbons	0.11
87	Chlorobenzene	Halohydrocarbons	0.35
88	Ethylbenzene	Aromatics	3.11

No.	Species	Chemical groups	MIR
89	m/p-Xylene	Aromatics	6.99
90	n-Nonane	Alkanes	1.03
91	o-Xylene	Aromatics	7.17
92	Styrene	Aromatics	1.70
93	Bromoform	Halohydrocarbons	–
94	Cumene	Aromatics	2.58
95	Symmetric tetrachloroethane	Halohydrocarbons	–
96	n-Propylbenzene	Aromatics	2.15
97	1-Ethyl-3-methylbenzene	Aromatics	6.70
98	p-Ethyltoluene	Aromatics	4.28
99	1,3,5-Trimethylbenzene	Aromatics	9.35
100	Decane	Alkanes	0.93
101	1-Ethyl-2-methylbenzene	Aromatics	5.33
102	Benzaldehyde	OVOCs	-0.33
103	1,2,4-Trimethylbenzene	Aromatics	7.88
104	1,3-Dichlorobenzene	Halohydrocarbons	–
105	p-Dichlorobenzene	Halohydrocarbons	0.20
106	1,2,3-Trimethylbenzene	Aromatics	9.86
107	Chlorotoluene	Halohydrocarbons	–
108	1,3-Diethylbenzene	Aromatics	6.30
109	o-Dichlorobenzene	Halohydrocarbons	0.20
110	p-Diethylbenzene	Aromatics	4.18
111	Undecane	Alkanes	0.85
112	3-Methylbenzaldehyde	OVOCs	-0.29
113	Dodecane	Alkanes	0.79
114	1,2,4-Trichlorobenzene	Halohydrocarbons	–
115	1,1,2,3,4,4-Hexachloro-1,3-butadiene	Halohydrocarbons	–
116	Naphthalene	Aromatics	3.13
117	Formaldehyde	OVOCs	7.16
118	Glyoxal	OVOCs	8.84
119	Cyclohexanone	OVOCs	1.66
120	Isovaleraldehyde	OVOCs	4.69
121	p-Methylbenzaldehyde	OVOCs	-0.29
122	Methylglyoxal (Pyruvaldehyde)	OVOCs	11.20
123	2,5-Dimethylbenzaldehyde	OVOCs	-0.26
124	Heptanal	OVOCs	3.55
125	Octanal	OVOCs	3.06
126	Nonaldehyde	OVOCs	–
127	Decanal	OVOCs	–

Table S3. Source profile of aircraft emissions.

Numbers	Species	Chemical groups	Concentration ± standard deviation (μg m^{-3})
1	Ethane	Alkanes	1.45 ± 0.24
2	Ethylene	Alkenes	1.24 ± 0.09
3	Propane	Alkanes	6.13 ± 0.41
4	Difluorodichloromethane	Halohydrocarbons	2.42 ± 0.03
5	Acrylic	Alkenes	0.21 ± 0.06
6	Isobutane	Alkanes	1.6 ± 0.37
7	n-Butane	Alkanes	2.44 ± 0.89
8	Acetylene	Alkynes	1.28 ± 0.07
9	trans-2-Butene	Alkenes	0.05 ± 0.02
10	cis-2-Butene	Alkenes	0.28 ± 0.06
11	n-Butene	Alkenes	0.1 ± 0.02
12	1,1,2,2-Tetrafluoro-1,2-dichloroethane	Halohydrocarbons	0.76 ± 0.06
13	Methyl chloride	Halohydrocarbons	–
14	Vinyl Chloride	Halohydrocarbons	–
15	Butadiene	Alkenes	0.21 ± 0.1
16	Acetaldehyde	OVOCs	3.61 ± 0.83
17	Methyl bromide	Halohydrocarbons	0.07 ± 0
18	Ethyl chloride	Halohydrocarbons	0.05 ± 0.02
19	Isopentane	Alkanes	1.08 ± 0.48
20	Monofluorotrichloromethane	Halohydrocarbons	1.01 ± 0.24
21	1-Pentene	Alkenes	0.06 ± 0.02
22	n-Pentane	Alkanes	1.24 ± 0.52
23	trans-2-Pentene	Alkenes	0.09 ± 0.05
24	Isoprene	Alkenes	0.61 ± 0.33
25	cis-2-Pentene	Alkenes	0.01 ± 0
26	Ethanol	OVOCs	1.76 ± 1.12
27	Acrolein	OVOCs	0.14 ± 0.04
28	Propionaldehyde	OVOCs	24.44 ± 6.87
29	1,1-Dichloroethylene	Halohydrocarbons	0.01 ± 0
30	1,2,2-Trifluoro-1,1,2-trichloroethane	Halohydrocarbons	0.75 ± 0.02
31	2,2-Dimethylbutane	Alkanes	0.13 ± 0.05
32	Acetone	OVOCs	19.47 ± 5.47
33	Carbon disulfide	Organic sulfur	–
34	Isopropyl alcohol	OVOCs	2.23 ± 1.42
35	Dichloromethane	Halohydrocarbons	12.66 ± 2.84
36	2,3-Dimethylbutane	Alkanes	0.2 ± 0.06
37	2-Methylpentane	Alkanes	0.89 ± 0.3
38	Cyclopentane	Alkanes	0.2 ± 0.05
39	trans-1,2-Dichloroethylene	Halohydrocarbons	0.01 ± 0.01
40	3-Methylpentane	Alkanes	0.37 ± 0.14
41	Methyl tert-butyl ether	OVOCs	0.28 ± 0.07
42	1-Hexene	Alkenes	0.03 ± 0

Numbers	Species	Chemical groups	Concentration ± standard deviation (μg m^{-3})
43	n-Hexane	Alkanes	1.97 ± 0.63
44	Methacrolein	OVOCs	0.19 ± 0.11
45	1,1-Dichloroethane	Halohydrocarbons	0.07 ± 0.02
46	Vinyl acetate	OVOCs	0.02 ± 0.01
47	2,4-Dimethylpentane	Alkanes	4.01 ± 1.4
48	n-Butyraldehyde	OVOCs	0.82 ± 0.35
49	Methylcyclopentane	Alkanes	0.16 ± 0.06
50	cis-1,2-dichloroethylene	Halohydrocarbons	0.01 ± 0
51	2-Butanone	OVOCs	3.46 ± 1.45
52	Ethyl acetate	OVOCs	7.13 ± 3.72
53	Chloroform	Halohydrocarbons	4.55 ± 1.91
54	Tetrahydrofuran	OVOCs	0.12 ± 0.07
55	1,1,1-Trichloroethane	Halohydrocarbons	0.01 ± 0
56	2-Methylhexane	Alkanes	0.23 ± 0.11
57	Cyclohexane	Alkanes	0.12 ± 0.06
58	2,3-Dimethylpentane	Alkanes	0.19 ± 0.1
59	Carbon tetrachloride	Halohydrocarbons	0.65 ± 0.02
60	3-Methylhexane	Alkanes	0.2 ± 0.13
61	Benzene	Aromatics	1.3 ± 0.15
62	1,2-Dichloroethane	Halohydrocarbons	7.61 ± 1.22
63	2,2,4-Trimethylpentane	Alkanes	0.18 ± 0.06
64	n-Heptane	Alkanes	0.42 ± 0.25
65	Crotonaldehyde	OVOCs	0.31 ± 0.18
66	Trichlorethylene	Halohydrocarbons	0.17 ± 0.1
67	Methylcyclohexane	Alkanes	0.08 ± 0.04
68	1,2-Dichloropropane	Halohydrocarbons	0.8 ± 0.1
69	Valeraldehyde	OVOCs	0.34 ± 0.09
70	Methyl methacrylate	OVOCs	0.06 ± 0.03
71	1,4-Dioxane	OVOCs	0.01 ± 0
72	Bromodichloromethane	Halohydrocarbons	0.03 ± 0
73	2,3,4-Trimethylpentane	Alkanes	0.04 ± 0.02
74	2-Methylheptane	Alkanes	0.03 ± 0.01
75	trans-1,3-Dichloro-1-propene	Halohydrocarbons	0.01 ± 0.01
76	3-Methylheptane	Alkanes	0.03 ± 0.01
77	4-Methyl-2-pentanone	OVOCs	0.26 ± 0.14
78	Toluene	Aromatics	3 ± 1.56
79	n-Octane	Alkanes	0.07 ± 0.02
80	cis-1,3-Dichloro-1-propene	Halohydrocarbons	0.01 ± 0.01
81	1,1,2-Trichloroethane	Halohydrocarbons	0.11 ± 0.03
82	Tetrachlorethylene	Halohydrocarbons	0.27 ± 0.15
83	2-Hexanone	OVOCs	1.38 ± 0.24
84	Dibromochloromethane	Halohydrocarbons	0.03 ± 0.01
85	Hexanal	OVOCs	1.14 ± 0.25
86	1,2-Dibromoethane	Halohydrocarbons	0.03 ± 0.02

Numbers	Species	Chemical groups	Concentration ± standard deviation (μg m^{-3})
87	Chlorobenzene	Halohydrocarbons	0.05 ± 0.02
88	Ethylbenzene	Aromatics	0.43 ± 0.1
89	m/p-Xylene	Aromatics	1.11 ± 0.35
90	n-Nonane	Alkanes	0.05 ± 0
91	o-Xylene	Aromatics	0.4 ± 0.11
92	Styrene	Aromatics	0.08 ± 0.02
93	Bromoform	Halohydrocarbons	0.07 ± 0.03
94	Cumene	Aromatics	0.02 ± 0
95	Symmetric tetrachloroethane	Halohydrocarbons	0.05 ± 0.03
96	n-Propylbenzene	Aromatics	0.04 ± 0.02
97	1-Ethyl-3-methylbenzene	Aromatics	0.09 ± 0.07
98	p-Ethyltoluene	Aromatics	0.05 ± 0.04
99	1,3,5-Trimethylbenzene	Aromatics	0.04 ± 0.03
100	Decane	Alkanes	0.04 ± 0.01
101	1-Ethyl-2-methylbenzene	Aromatics	0.05 ± 0.03
102	Benzaldehyde	OVOCs	0.23 ± 0.04
103	1,2,4-Trimethylbenzene	Aromatics	0.15 ± 0.03
104	1,3-Dichlorobenzene	Halohydrocarbons	0.05 ± 0.03
105	p-Dichlorobenzene	Halohydrocarbons	0.07 ± 0.04
106	1,2,3-Trimethylbenzene	Aromatics	0.05 ± 0.04
107	Chlorotoluene	Halohydrocarbons	0.03 ± 0.02
108	1,3-Diethylbenzene	Aromatics	0.02 ± 0.01
109	o-Dichlorobenzene	Halohydrocarbons	0.05 ± 0.03
110	p-Diethylbenzene	Aromatics	0.02 ± 0.01
111	Undecane	Alkanes	–
112	3-Methylbenzaldehyde	OVOCs	–
113	Dodecane	Alkanes	–
114	1,2,4-Trichlorobenzene	Halohydrocarbons	–
115	1,1,2,3,4,4-Hexachloro-1,3-butadiene	Halohydrocarbons	–
116	Naphthalene	Aromatics	–
117	Formaldehyde	OVOCs	6.37 ± 1.24
118	Glyoxal	OVOCs	1.82 ± 0.26
119	Cyclohexanone	OVOCs	1.25 ± 0.77
120	Isovaleraldehyde	OVOCs	0.81 ± 0.13
121	p-Methylbenzaldehyde	OVOCs	–
122	Methylglyoxal (Pyruvaldehyde)	OVOCs	1.82 ± 0.34
123	2,5-Dimethylbenzaldehyde	OVOCs	0.78
124	Heptanal	OVOCs	3.79 ± 0.49
125	Octanal	OVOCs	1.55 ± 0.74
126	Nonaldehyde	OVOCs	3.93 ± 1.05
127	Decanal	OVOCs	3.81 ± 1.01
